# The Effect of Environment on Ventral Abdominal Temperature in Five Tiger Beetle Species (Coleoptera: Cicindelidae)

**DOI:** 10.3390/biology15080599

**Published:** 2026-04-10

**Authors:** John L. Bowley, Leon G. Higley, Robert K. D. Peterson

**Affiliations:** 1 Department of Land Resources and Environmental Sciences, Montana State University, Bozeman, MT 59717, USA; john.bowley@nebraska.gov; 2 School of Natural Resources, University of Nebraska, Lincoln, NE 68198, USA; lhigley1@unl.edu

**Keywords:** thermophile, extremophile, ecophysiology

## Abstract

Recent work demonstrated that *Cicindelidia hemorrhagica* (LeConte) inhabiting geothermal springs in Yellowstone National Park (YNP) possess morphological traits that reduce internal heat load when exposed to bottom-up thermal stress. To investigate whether this pattern extends to other tiger beetle species occupying diverse environments, we quantified the internal abdominal temperatures of six species differing in habitat preference and putative thermal adaptation. Using a water-bath system that simulated surface heating, we compared the temperature differential (ΔT) between beetle-loaded and bare thermocouples across multiple temperatures. Linear mixed-effects models were used to evaluate the influence of location and species on internal temperature. Across all experimental temperatures, *C. hemorrhagica* exhibited the greatest ΔT values, indicating the lowest internal temperatures relative to the thermal environment, regardless of whether individuals originated from YNP or non-thermal Idaho habitats. In contrast, the warm-resilient *Cicindela repanda* (Dejean) and non-warm-adapted *C. longilabris* (Say) showed the smallest ΔT values and therefore the highest internal temperatures. Ventral abdominal coloration—ranging from bright red (*C. sedecimpunctata* (Klug)) to dark blue-green (*C. oregona* (Dejean))—did not correlate with internal temperatures, suggesting that it is a poor predictor of heat absorbance or reflectance under bottom-up heat exposure. These results indicate that *C. hemorrhagica* is uniquely effective at limiting internal heat gain from surface heating, and that it may possess a preadaptive morphological mechanism facilitating thermal resistance in geothermal habitats.

## Simple Summary

Thermal tolerance in tiger beetles (Coleoptera: Cicindelidae) varies widely across species and habitats, yet the mechanisms underlying these differences remain poorly understood. *Cicindelidia hemorrhagica* (LeConte) inhabiting geothermal springs in Yellowstone National Park (YNP) possess morphological traits that reduce internal heat load when exposed to bottom-up thermal stress. To investigate whether this pattern extends to other tiger beetle species, we quantified the internal abdominal temperatures of six species differing in habitat preference and putative thermal adaptation. Using a water-bath system that simulated surface heating, we compared the temperature differential between beetle-loaded and bare thermocouples across multiple temperatures. Across all experimental temperatures, *C. hemorrhagica* exhibited the greatest temperature differential, indicating the lowest internal temperatures relative to the thermal environment. In contrast, the warm-resilient *Cicindela repanda* (Dejean) and non-warm-adapted *C. longilabris* (Say) showed the smallest ΔT values and therefore the highest internal temperatures. These results indicate that *C. hemorrhagica* is uniquely effective at limiting internal heat gain from surface heating.

## 1. Introduction

We previously characterized internal ventral abdominal temperatures and heat resistance in adult wetsalts tiger beetles, *Cicindelidia hemorrhagica* (LeConte), in Yellowstone National Park (YNP) [1]. Adults in YNP are associated exclusively with hot springs, and individuals reflect more heat from the ventral surface of their abdomens and behave differently than *C. hemorrhagica* adults living in non-hot springs environments in Idaho. Although *C. hemorrhagica* is a warm-adapted tiger beetle [2], the population in YNP seem to have evolved increased heat reflectance on the ventral portions of their abdomens. This increased heat reflectance seems to be caused by a heatshield-like physical feature which is part of the exoskeleton’s ventral abdominal plate; this is consistent with the fact that they experience bottom-up heat from the geothermal springs [1,3,4,5,6].

This discovery leads to additional questions about variation in ventral heat reflectance and absorbance among tiger beetle species occupying diverse environments. The ability to tolerate and resist hot environments, including those with high insolation and high surface temperatures, has led some tiger beetle species to exhibit very high lethal temperature maxima compared to other insects [1,2,7,8].

The ability to withstand high temperature is a key feature for cicindelid biology, as many species occupy high-temperature niches. The ability to forage and survive at high surface temperatures opens ecological opportunities for adapted beetles. In particular, success in predation and scavenging will increase as potential prey items are stunned by high temperature. Such foraging on stunned prey seems to be a key strategy for the success of *C. hemorrhagica* in geothermal springs [1].

In this study, we address the following questions: Does YNP *C. hemorrhagica* reflect the most heat from its abdomen compared to several other species? Do species that have adapted to these warmer environments and have resided in them for multiple generations have lower internal body temperatures in the face of high ambient temperatures compared to species which are not adapted to warmer temperatures, or which have not been found present in warmer environments for longer than the length of one generation? If so, are there morphological attributes, including abdomen color, that distinguish warm-adapted species from non-warm-adapted species?

## 2. Materials and Methods

We collected five species for internal-temperature comparison experiments (Table 1). The species were chosen based on availability and on exhibition of variation in adult temperature and in habitat preference. Two species are considered warm-adapted, one species is considered non-warm-adapted, and two are considered warm-resilient [2] (Table 1). Although these terms are not objective or well defined, warm-adapted means that the species prefers hot temperatures, often spending considerable time in full sun. Warm-resilient means that the species can spend time in hot, dry conditions but has more diversity in its habitats than a warm-adapted species. Non-warm-adapted means that the species avoids hot, dry conditions (Table 1).

The putatively warm-adapted *C. hemorrhagica* were obtained from three separate populations. Adults were collected from Yellowstone National Park (YNP) at Dragon Spring and Rabbit Creek (coordinates not publicly available, per NPS-YNP research permit conditions). Adults were also collected from a salt flat located approximately 26 km south of Mountain Home, Idaho (42.9354, −115.7504). All specimens were collected using 38 cm aerial sweep nets (Gemplers, Janesville, WI, USA) through “sneak and pounce” netting techniques. Once collected, individuals were placed in small plastic containers containing damp paper towels to prevent desiccation before freezing. A total of 16 specimens were collected from both YNP locations; these were collectively named “Park” to enable YNP and Idaho *C. hemorrhagica* populations to be distinguished. Specimens were collected in the months of July and August from 2019 to 2021.

Similar methods were used to collect the putatively warm-resilient species *Cicindela oregona* (Dejean) [2] in Glen Lake Rotary Park in Bozeman, Montana (45.7051, −111.0387). Specimens were collected in June and July of 2019 and 2020. Beetles were found along the northern public beach area of this location, and they were only present when conditions of minimal wind and direct sunlight on the sand were met. *Cicindela longilabris* (Say) (a non-warm-adapted species) [2] specimens were collected directly on the Olsen Creek Road Jeep Trail approximately 23 km northeast of Bozeman, Montana (45.8272, −110.7405). Members of this putatively non-warm-adapted species [2] were located directly on the trail in full sun under canopy cover and collected sporadically from May through August of 2020.

Specimens of the putatively warm-adapted *Cicindelidia sedecimpunctata* (Klug) [2] were collected near Portal, Arizona (31.8806, −109.2036) on collection trips during July 2020 by lab associates and brought back to Montana State University (MSU) using the same methodology previously described. The putatively warm-resilient *Cicindela repanda* [2] were collected during September 2021 near Rock Island, Illinois (41.5449, −90.4199) and shipped directly from associates at Augustana College and frozen upon arrival at MSU. Summaries of collected specimens are listed in Table 2.

To obtain a better understanding of the internal temperatures experienced by multiple species of the same genus that live in a variety of environments, we conducted a series of laboratory experiments focused on exposing each species to the same regulated environment. Approximately 16 specimens of *C. haemorrhagica* from YNP, 16 specimens of *C. haemorrhagica* from Idaho, 24 specimens of *C. oregona* from Glen Lake Rotary Park in Bozeman, MT, 10 specimens of *C. sedecimpunctata* from Illinois, 10 specimens of *C. repanda* from Portal, AZ, and 5 specimens of *C. longilabris* from Olson Creek Trail were collected for experimentation. All specimens were frozen upon arrival at MSU and stored in a −20 °C freezer. Dry weights were recorded for each beetle after each experiment.

**Figure 1:**
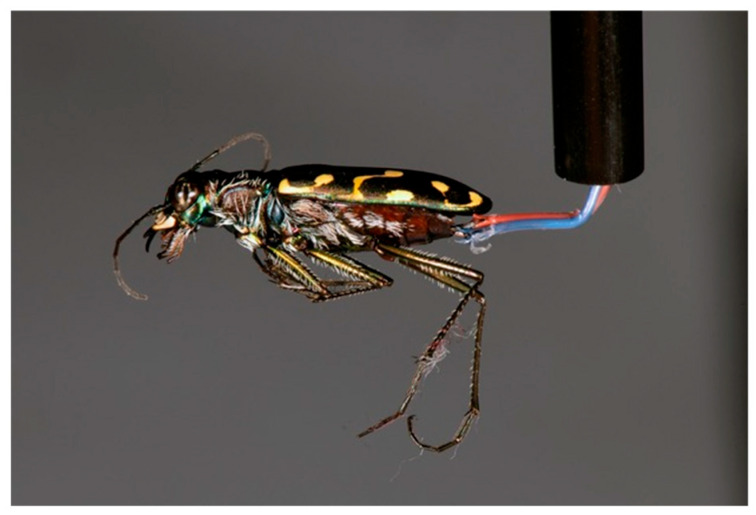
*Cicindelidia hemorrhagica* adult with a thermocouple inserted into its abdomen in preparation for water-bath experiment. Photo by RKD Peterson.

The methods for the experimental assay were nearly identical to those we presented in [1]. We prepared a heated water bath by filling a 12 L sous vide tub (Rubbermaid, Wooster, OH, USA) with approximately 8 L of water, and placed a Bluetooth Joule-branded sous vide (ChefSteps, Seattle, WA, USA) device inside the bath to regulate temperature. Thermocouples were produced using 24-American Wire Gauge thermocouple wire and T-type thermocouple connector plugs (OMEGA, Norwalk, CT, USA). Framework was made from 6 mm diameter plastic restaurant straws that were cut to 10 cm increments to keep the thermocouple wire straight. Wires threaded through the framework were suspended above the water bath at approximately 2 mm using a 2.5 cm thick × 5.1 cm wide wooden plank with notches carved 2.5 cm deep into the side of the board to hold the reinforced thermocouple wire [1]. The exposed thermocouple wires were bent at a 90° angle above the water bath to simulate beetle body length and height above the water bath perpendicular to the water’s surface. Any recorded temperatures were logged using the SD-947 thermometer/SD card data logger 4-channel model (REED, Wilmington, NC, USA). Using methodology that exhibited previous experimental success [1], we inserted the exposed end of the thermocouple wire into each beetle through the genital opening. The wire extended up the length of each beetle abdomen without compromising the structure of the exoskeleton or penetrating the thorax (Figure 1). Each wire was positioned at the approximate midline between the ventral and dorsal portions of the abdomen in each beetle. Later replicates of the experiment were conducted with legs removed from each individual to ensure the thermocouples were not exposed to any accumulating water that may travel up the legs and into the abdominal cavity where the thermocouple was recording temperature readings.

**Figure 2:**
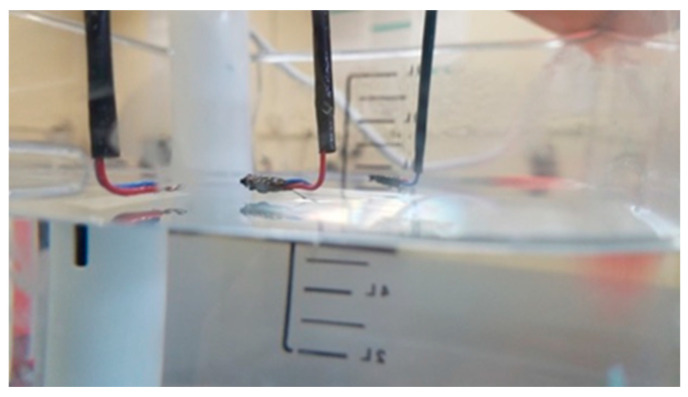
Recording *Cicindelidia hemorrhagica* adult internal temperatures. Thermocouple wires were suspended above the water bath in which the temperature was regulated by the sous vide (left) and recorded using the thermocouple logger/recorder. Photo by JL Bowley.

Beetles from each species were subject to multiple water-bath experiments at different temperatures. *Cicindelidia oregona*, *C. longilabris*, *C. sedecimpunctata*, and *C. repanda* were exposed to water bath temperatures of 50 °C, 55 °C, and 60 °C. Each replicate of the experiment consisted of one beetle-loaded thermocouple and one bare thermocouple suspended above the heated water bath using the wooden plank and framework material (Figure 2). Thermocouples were allowed 2 min to equilibrate before we began recording temperatures. Each replicate of the experiment had a recording period of 10 min, with recording of the temperature reading of each thermocouple taking place at 1 min intervals. The water bath temperatures for testing *C. hemorrhagica* from YNP and Idaho consisted of one beetle-loaded thermocouple from each population with a third bare thermocouple to continue to act as the control variable. The procedure for preparing these experiments was the same, but beetles were exposed to water bath temperatures of 45 °C, 50 °C, and 55 °C. Therefore, we were only able to use this species when comparing the results of the 50 °C and 55 °C experiments.

Because not every thermocouple displayed the same temperature measurement as the bare thermocouple control when measuring the ambient air temperature, we had to calibrate the thermocouples [1]. The control and the thermocouples used for adult beetles only differed by a maximum of 0.2 °C, so thermocouples were calibrated ±0.2 °C to account for the difference in measured temperature between the thermocouples and the bare thermocouple that was used as the control. Each thermocouple was measured against the control before recording for this reason.

### Data Analysis

We performed initial data visualizations and *t*-tests for each group using Microsoft Excel. The temperature values for each thermocouple-paired treatment with a beetle attached to it were directly compared to the bare thermocouple temperature reading for each minute of recording using the following equation:

∆*T* = *T_Bare_* − *T_Beetle_* (1)

where ∆*T* is the difference in temperature between the bare thermocouple and the thermocouple with the beetle. This value expresses the difference between the temperature readings for the thermocouple exposed to the open air of the heated water bath environment and the layer of reflective protection provided by the abdominal cavity. The ∆*T* values of each pairing were totaled and averaged according to the temperature of the water bath to produce one average ∆*T* per temperature per beetle. These average values were grouped together by species and used to calculate the standard deviation and standard error. All calculations were used to produce preliminary figures used in data visualization.

The data were reformatted for compatibility with RStudio (version 2026.01.1+403 for Windows) by grouping individual results by species, collection site, and then water bath temperatures with their respective ∆*T* values. Initial linear models produced in RStudio used ∆*T* as the variable measured against the confounding variables of location, dry weight of the individual, legs attached, sex, and air temperature. We determined that this model was inadequate to use in the analysis because each individual was subjected to multiple replicates of the experiment at different temperatures. Therefore, we produced a linear mixed effects model using the lme4 package in RStudio [9].

(2) Tiger Model1<−lm(DT ` Location+Weight+Legs+Sex, data=Beetle Data)

Results of this initial model suggested that weight and sex were not influential on internal temperatures experienced by the beetle-loaded thermocouples (*p*-values 0.7716 and 0.3591, respectively). Therefore, an additional model was produced excluding these variables. Leg presence was also excluded from this revised model because we had insufficient data to compare leg presence for each species (only *C. hemorrhagica* and *C. oregona*).

(3) Tiger Model Final<−lme(DT ` Location, Random=` 1 | Individual, Data=Beetle Data)

Our final model only measured the effects of species (labeled by collection location) on internal temperatures experienced by individuals subject to multiple water-bath experiments to simulate a hazardous environment heated from below by thermal waters (Table 3).

## 3. Results and Discussion

**Figure 3:**
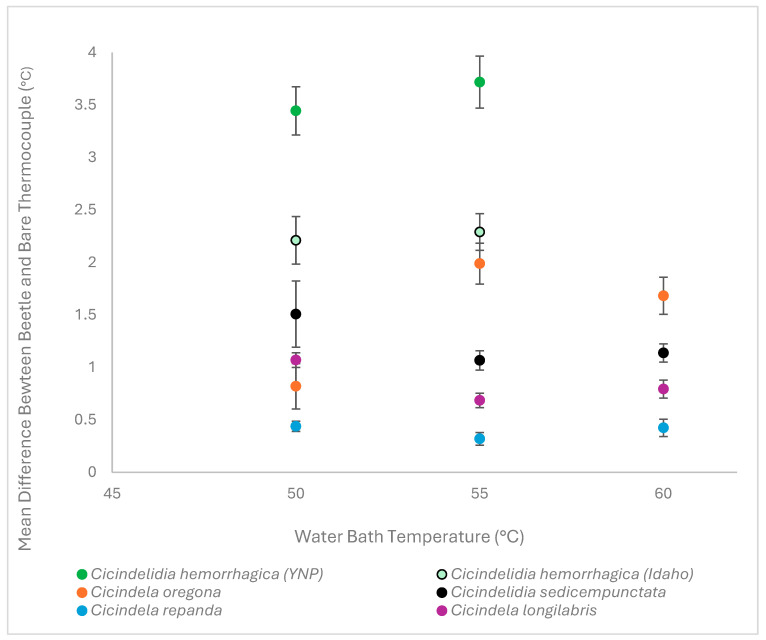
Mean differences in temperatures between thermocouples in beetle abdomens versus the bare thermocouple controls (∆T). Error bars consist of standard error values calculated from each species at the given water bath temperature.

The mean ∆*T* values suggest that *C. hemorrhagica* exhibited the greatest difference in internal temperatures compared to the bare thermocouple (Table 1, Figure 3). This means that *C. hemorrhagica* had the lowest internal abdominal temperatures compared to the other species. Conversely, *C. repanda* had the smallest difference over all water bath temperatures, and therefore it exhibited the highest internal temperatures (Table 4 and Table 5, Figure 3). However, for the other species the mean difference between internal beetle temperature and the bare thermocouple depended on water bath temperature (Table 5, Figure 3).

*Cicindelidia sedecimpunctata* is a warm-adapted desert species, closely related to *C. hemorrhagica*, and also has a bright red-to-orange abdomen. However, and surprisingly, *C. sedecimpunctata* was not the next species in order of greatest difference in internal temperatures compared to the thermocouple (Figure 3). This suggests that the bright-colored abdomen did not reflect more heat greater compared to the darker abdomens of other species.

Interestingly, the *C. oregona* measured in this study had navy blue to dark green abdomens yet had low internal temperatures (i.e., large differences between internal temperatures and bare thermocouple) (Figure 3), further suggesting that abdomen color is likely a poor predictor of internal temperature, heat absorbance, or heat reflectance.

The non-warm-adapted species, *C. longilabris*, had the second smallest difference between internal temperatures and the bare thermocouple (Figure 3). Specimens measured in this study had dark blue-green abdomens. The *C. repanda* measured in this study had the smallest difference between internal temperatures and the bare thermocouple and had blue-green abdomens (i.e., the highest internal temperatures). *Cicindela repanda* (warm-resilient) and *C. longilabris* (non-warm-adapted) consistently had higher internal temperatures compared to *C. hemorrhagica* and *C. sedecimpunctata*, the species that are found in hot springs and in desert-like or high sun exposure environments (Table 1). Specimens frozen for preservation in preparation for experimentation may have influenced internal temperature readings by the thawing of viscera or altering internal structures (but see [1]). However, these experiments were focused on measuring the structural reflectivity in the abdomen of each species. Therefore, the desiccation of each specimen before testing was essential to ensure that the thermocouples inserted into each beetle experienced similar microclimates.

We previously showed that *C. hemorrhagica* from YNP hot springs had statistically greater differences in internal temperatures compared to *C. hemorrhagica* from a non-hot springs environment in Idaho (Figure 3) [1]. Here, *C. hemorrhagica* (regardless of location) had the lowest internal temperatures compared to all other species evaluated.

Therefore, *C. hemorrhagica* may be preadapted to reflect more heat from its ventral surface than the other species we evaluated. This study is not unique in analyzing microstructures that result in reduced experienced temperatures on individual insect specimens. Thermotolerant species such as the Saharan silver ant, *Cataglyphis bombycina* (Hymenoptera: Formicidae), *Neoceramyx gigas* (Coleoptera: Cerambycidae), and *Goliathus goliatus* (Coleoptera: Scarabaeidae) all possess differing physical structures that aid in heat reflectance to make them more tolerant of extreme environmental conditions [10,11,12]. This hypothesis is supported by the observation that individuals of the Idaho *C. hemorrhagica* species that is not associated with hot springs had significantly lower internal temperatures in the presence of bottom-up heating compared to all other species evaluated (Figure 3). Consequently, this preadaptation may have been selected for by YNP geothermal springs environments, resulting in the behavioral and morphological adaptations observed in *C. hemorrhagica* in YNP [1,4,5,6].

## 4. Conclusions

Across all manipulated temperatures measured, *C. hemorrhagica* exhibited the greatest ΔT values, indicating the lowest internal temperatures relative to the thermal environment stress. Conversely, the warm-resilient *Cicindela repanda* and non-warm-adapted *C. longilabris* had the smallest ΔT values, indicating the highest internal temperatures. Ventral abdominal coloration, from bright red (*C. sedecimpunctata*) to dark blue-green (*C. oregona*), did not correlate with internal temperatures, suggesting that coloration may be a poor predictor of heat absorbance or reflectance from bottom-up heat exposure. These results indicate that *C. hemorrhagica* is effective at limiting internal heat gain from surface heating stress and may possess a preadaptive morphological mechanism facilitating thermal resistance in geothermal habitats.

Because we used a water bath with bottom-up heat in our experiments, we can only draw conclusions about internal temperatures based on surface heating. It is possible that some of the cicindelid species which do not experience high surface heat may be adapted more to top-down heat from insolation. In this case, future research is needed that evaluates internal temperatures from top-down “dry” heat.

## Figures and Tables

**Table 1:** Biological and ecological information for each species that was collected and used for experiments.

Species	Dorsal/Ventral Color	Range	Habitat	Temperature Adapted
*Cicindelidia hemorrhagica* (LeConte)	Dorsal dark coppery brown to black. Ventral is dark purple to copper with bright red abdomen	CA to WA to East WY and south to west TX	Found residing near water features: Ponds, lakes, reservoirs, rivers, springs, salt flats, and ocean beaches	Considered warm adapted to environments with full sun present
*Cicindela oregona* (Dejean)	Dorsal blue-black to green. Ventral of southern populations metallic blue to purple with upper sides showing coppery to green blue or purple—intermediate Montana population found was predominantly coppery bronze dorsal face and metallic blue green ventral face.	Wide range—Western region of North America	Found primarily along ocean beaches or desert water features. Can be found along water’s edge in cooler moist areas further inland.	Considered resilient and can live in hot dry, desert regions
*Cicindela repanda* (Dejean)	Dorsal bronze-brown to reddish-brown. Ventral blue-green to copper	Widespread Continental US and CA	Wide variety but typically found along sandy environments adjacent to water sources. Also occurs in dry, sandy areas above beaches. Less common in the west.	Considered resilient and can live in hot dry, desert regions
*Cicindelidia sedecimpunctata* (Klug)	Dorsal light to dark brown. Ventral dark copper/blue with bright orange/red abdomen	Arizona to southern Mexico	Desert areas with streams to lakes and grassy marshlands, mud flats in the extreme southwest.	Warm adapted to desert environments—will move up to higher altitudes after major rain events in lowland areas
*Cicindela longilabris* (Say)	Dorsal dull black to coppery brown to bright green. Ventral dark metallic green to blue-purple	Boreal zone from Canada and Maine into Northwestern Canada, stretching through the Rockies into Montana and Idaho	Sandy/gravelly soils through open walkways in high altitude coniferous forests	Not considered warm adapted—present in high altitude environments

**Table 2:** Condensed table showing where each species was collected and how many individuals were captured at each location that were used for experimentation.

Species	Location of Collection	Number of Specimens Collected
*Cicindelidia hemorrhagica*	Yellowstone National Park	16
*Cicindelidia hemorrhagica*	Idaho	16
*Cicindelidia sedecimpunctata*	Illinois	10
*Cicindela oregona*	Montana	24
*Cicindela repanda*	Arizona	10
*Cicindela longilabris*	Montana	5

**Table 3:** Variables used in the linear models developed in the analysis and their respective abbreviations.

Variable	Abbreviation
Linear Model	lm
Linear Mixed Effects Model	lme
Difference in Temperature	DT (∆T)
Location in which specimens were collected	Location
Dry Weight of Beetle	Weight
Legs present on the individual	Legs
Sex of individual	Sex

**Table 4:** Summary statistics of the linear fixed-effects model comparing internal temperatures experienced by individuals at different water bath temperatures from different locations. Degrees of freedom in the model refer to the intercept of the model compared to the number of replicates conducted at different water temperatures performed on each beetle in addition to each beetle tested in experimentation. *P*-values suggest that collection location and, potentially, species have a strong influence on experienced internal temperatures when subjected to environments heated from below.

Location	Latitude, Longitude *	Species	∆T (°C) **	SE (°C) ***	df	t-Value	*p*-Value
Portal, Arizona (Intercept)	31.8806, −109.2036	*Cicindelidia sedecimpunctata*	0.39	0.19	186	2.1122	0.0360
Olsen Creek Road, Montana	45.8272, −110.7405	*Cicindela longilabris*	0.46	0.32	75	1.4072	0.1635
Bozeman, Montana	45.7051, −111.0387	*Cicindela oregona*	1.05	0.23	75	4.6417	<0.0001
Rock Island, Illinois	41.5449, −90.4199	*Cicindela repanda*	0.84	0.26	75	3.1902	0.0021
Snake River, Idaho	42.9354, −115.7504	*Cicindelidia hemorrhagica*	1.57	0.23	75	6.8715	<0.0001
Yellowstone National Park		*Cicindelidia hemorrhagica*	2.63	0.23	75	11.4700	<0.0001

* Lat/Long information protected per YNP research permit stipulation. ** The difference between internal body temperature and bare thermocouple. *** Standard error of the mean.

**Table 5:** The average difference in temperature between internal body temperatures and bare thermocouples measuring exposed ambient air temperatures for each species in the experiment. Standard error values for each of the respective temperatures used to produce error bars in Figure 3 are also provided.

Species	Average ∆T 50	Average ∆T 55	Average ∆T 60	Standard Error ∆T 55	Standard Error ∆T 55	Standard Error ∆T 60
*Cicindelidia hemorrhagica (YNP)*	3.445	3.718125		0.2299609	0.2477827	
*Cicindelidia hemorrhagica (ID)*	2.210625	2.290625		0.2264355	0.1739937	
*Cicindelidia sedicempunctata*	1.508	1.067	1.13	0.3153817	0.0921634	0.0875077
*Cicindela oregona*	0.8208333	1.9895	1.683	0.2163434	0.1950432	0.1768628
*Cicindela repanda*	0.439	0.32	0.425	0.0485067	0.0601166	0.0821493
*Cicindela longilabrus*	1.0702	0.686	0.794	0.0695416	0.0688535	0.0864222

## Data Availability

Data are available on request.
